# Adherence to international guidelines in neurocritical care of cervical traumatic spinal cord injury-a retrospective study

**DOI:** 10.1016/j.bas.2024.102821

**Published:** 2024-04-21

**Authors:** Fredrika Rask, Erik Uvelius, Niklas Marklund

**Affiliations:** a Department of Clinical Sciences Lund, Neurosurgery, Lund University, Lund, Sweden; b Department of Clinical Sciences Lund, Neurosurgery, Lund University, Skåne University Hospital, Lund, Sweden

**Keywords:** Spinal cord injury, Monitoring, Neurocritical care, Mean arterial blood pressure, Outcome

## Abstract

**Introduction:**

The American Association of Neurologic Surgeons guidelines on the management of traumatic spinal cord injury (SCI), updated in 2013, focus on spinal cord perfusion, early decompressive surgery, and venous thromboembolism (VTE) prophylaxis to improve neurological outcome.

**Research question:**

How neurocritical care and initial management have changed with the implementation of updated management guidelines, focusing on guidelines adherence and neurological outcome.

**Material and methods:**

Systemic physiological variables, time to neurosurgical treatment and VTE prophylaxis, and neurological outcome, were retrospectively collected from adult patients treated for cervical SCI 2001–2021.

**Results:**

Fifty-two patients were included. Mean arterial blood pressure (MAP) was significantly higher after 2013 (86±9.9 mmHg) when compared to before 2013 (79±9.9 mmHg), p = 0.041. Median time to surgery was 41 h before, and 20 h after 2013 (p = 0.029). Time to VTE prophylaxis was six days before and four days after 2013. Most neurocritical care complications were less commonly observed after 2013. Despite improved adherence to treatment goals, 44 % of MAP levels were below target, and 33% of patients were operated beyond 24h post-injury. Neurological outcome was not improved after implementation of the revised guidelines.

**Discussion and conclusion:**

While implementation of the revised 2013 guidelines improved most aspects of the acute SCI management, many guideline targets were not met in a large subset of patients. Strict adherence to the acute neurocritical management goals, and early surgical treatment, is likely crucial when aiming to improve SCI outcome.

## Highlights

• Guidelines in SCI care were updated in 2013.
• Implementing guidelines improved MAP, VTE prophylaxis, and time to surgery without improved outcome.
• In a large subset of patients, MAP targets were not reached and surgery was performed beyond 24 h post-injury.
• Stringent adherence to guideline treatment goals is warranted to enable improved outcome.

## 1 Introduction

A traumatic spinal cord injury (SCI) is a devastating event, often causing various degrees of paralysis, sensory loss, pain, and autonomic dysfunction with life-changing functional consequences to the patient. The neural injury process can be divided into two phases. The primary, direct injury, is an immediate mechanical disruption of the spinal cord whereas the secondary insult is caused by complex cellular cascades involving inflammation, edema, and loss of cellular stability. In addition, impaired spinal cord perfusion is common due to e.g. loss of neural control of blood pressure and compression of the spinal cord (Adegeest et al., 2023; Ahuja et al., 2017; Evaniew et al., 2024). These secondary mechanisms peak during the first week post-injury (Sekhon and Fehlings, 2001). Preserving neurological function is critical as even a discrete deterioration of the tissue injury, especially at the cervical level, may severely affect the patients' independence. To date, since the evidence supporting early methylprednisolone treatment is disputed (Liu et al., 2019; Geisler et al., 2023), there are no pharmacological treatment options with proven clinical benefits available for SCI patients. An optimal initial evaluation and medical management could potentially minimize the extent of secondary injury (Evaniew et al., 2024; Hachem et al., 2017; Kwon et al., 2024; Fehlings et al., 2017). However, treatment options are scarce and there is limited scientific support for the neurocritical care and practical management of acute SCI (Hachem et al., 2017; Fehlings et al., 2017).

Current management guidelines for acute spinal cord injuries were updated in 2013 by AANS (American Association of Neurological Surgeons) and CNS (Congress of Neurological Surgeons)(Walters et al., 2013). These guidelines especially highlight the importance of maintaining spinal cord perfusion during the first week post-injury, early decompressive surgery, and early initiation of venous thromboembolism (VTE) prophylaxis. The present retrospective study aimed to review how neurocritical care and initial management of acute cervical SCI changed with the introduction of the 2013 guidelines and to evaluate the adherence to SCI guidelines at our neurosurgical center.

## 2 Method and materials

### 2.1 Study population

**Fig. 1:**
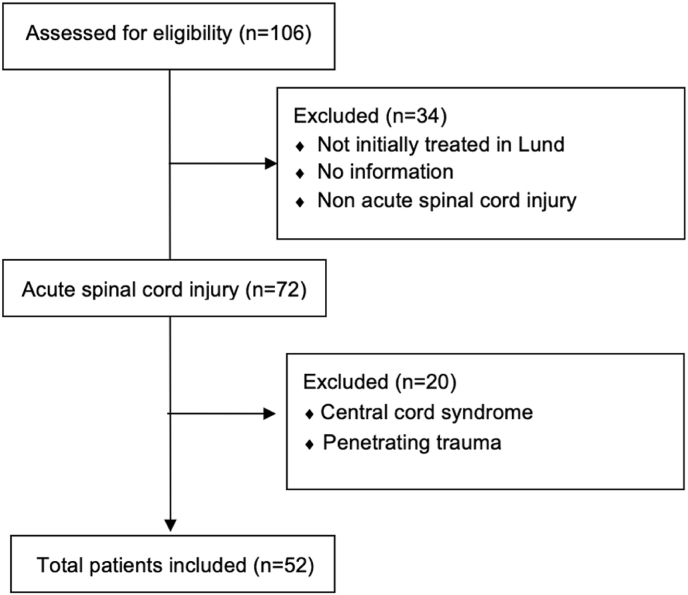
Consort flowchart of inclusion.

The study population included adult patients with cervical SCI treated at the Department of Neurosurgery, Skåne University Hospital, Lund, Sweden, between 2001 and 2021. The hospital is a tertiary trauma center for 1.9 million inhabitants. Patients with non-traumatic injuries or penetrating trauma were excluded. Additionally, due to the controversies surrounding on the optimal treatment of central cord syndrome both regarding pathophysiology and timing of intervention, patients with central cord syndrome were excluded (Badhiwala et al., 2020, 2022). Among 106 reviewed patients, 52 patients were included. Inclusions and exclusions are presented in Fig. 1. Ethical approval was obtained from the Swedish Ethical Review Authority (Dnr, 2017/469) and institutional review approval by Region Skåne (KvB). The study was conducted according to the Declaration of Helsinki.

### 2.2 Data collection

Hourly data were extracted from patients’ files using a monitoring account in Melior (Cerner, Kansas City, USA) and the critical care platform IntelliSpace Critical Care and Anesthesia (Philips Medizin Systeme, Böblingen, Germany). The collected variables were age, sex, year of injury, trauma mechanism, hours with temperatures higher than 38, 39, and 40 °C, days in neurocritical care, and mortality. Mean arterial blood pressure (MAP), systolic blood pressure, and saturation every hour during the initial seven days in neurocritical care and which ionotropic treatment and fluids (colloids, crystalloids, and blood) were administrated. The timing of surgical intervention and surgical approach were registered. The selection of surgical approach and technique was at the discretion of the treating surgeon. Duroplasty, although a recent potential refinement of the neurosurgical treatment in selected SCI patients, was not used in any of the patients in the present study. Complications (deep venous thrombosis, pulmonary embolism, urinary tract complications, pressure ulcers, wound infection, and pneumonia) were noted. The type and timing of VTE prophylaxis and whether corticosteroids were given were also included. Neurological status according to the American Spinal Injury Association (ASIA) International Standards for Neurological Classification of Spinal Cord Injury (ISNCSCI) worksheet graded patients by the ASIA Impairment Scale (AIS), at arrival, after neurocritical care and after rehabilitation. Outcome was assessed through the difference in AIS grade at arrival and three months after the injury. Improvement was divided into no improvement, one, two, or three grades of improvement. In cases where preoperative magnetic resonance imaging (MRI) was performed, the images were analyzed regarding medullary compression, the anterior-posterior diameter of the medulla, and the length of medullary edema.

### 2.3 Statistical analysis

Statistical analyses were performed using Microsoft Excel version 16.52 and IBM SPSS Statistics 27. Normally distributed data were presented as mean ( ± standard deviation, SD) and analyzed using *t*-test. Non-parametric variables and skewed data were presented as median (range) and analyzed using the Mann-Whitney *U* test. One-way ANOVA was used with multiple variables to analyze mean MAP or mean spinal cord diameter between the four groups of AIS improvement. Significant results were analyzed using post-hoc least significant difference analysis. Kruskal-Wallis test was used to analyze differences in non-normally distributed values between multiple groups. P-values <0.05 were considered significant.

## 3 Results

The study population consisted of 52 patients with a 3:1 ratio between men and women. All patients had cervical injuries and 35 (67%) had AIS grade of A-B, i.e. motor complete injuries. The mean age was 53 years, ranging from 18 to 87 years. Transport accidents were the most common cause of injury (46%). The median time in the neurointensive care unit was five days. Corticosteroid treatment was initiated in 32% of patients injured before 2013 and in 7% after 2013 Table 1.

**Table 1:** Patient characteristics.

	TOTAL (N = 52)	BEFORE 2013 (N = 37)	AFTER 2013 (N = 15)
**SEX**			
- WOMEN, N (%)	14 (27)	9 (24)	5 (33)
- MEN, N (%)	38 (73)	28 (76)	10 (67)
**AGE, MEAN (SD, RANGE)**	53 (18, 18–87)	54 (17, 20–87)	51 (21, 18–85)
**TRAUMA MECHANISM**			
- TRANSPORT ACCIDENT, N (%)	24 (46)	20 (54)	4 (27)
- SPORT ACCIDENT, N (%)	4 (8)	–	4 (27)
- FALL, N (%)	23 (44)	17 (46)	6 (40)
- OTHER, N (%)	1 (2)	–	1 (6)
**TIME TO SURGERY, HOURS, MEDIAN (IQR)**	40 (72)	41 (74)	20 (49)
**TIME TO VTE PROPHYLAXIS, DAYS, MEAN (SD)**	5 (3.8)	6 (4)	4 (3)
**DEEP VENOUS THROMBOSIS, N (%)**	3 (6)	2 (5)	1 (7)
**PULMONARY EMBOLISM, N (%)**	3 (6)	3 (8)	0 (0)
**CORTICOSTEROID TREATMENT, N (%)**	13 (25)	12 (32)	1 (7)
**FEVER**			
- HOURS >38 °C, N	624	394 (6)	230 (9)
- HOURS >39 °C, N	128	81 (1.3)	47 (2)
- HOURS >40 °C, N	19	13 (0.2)	6 (0.2)
**INFECTION**			
- URINARY TRACT INFECTION, N (%)	21 (40)	13 (35)	8 (53)
- PNEUMONIA, N (%)	16 (31)	12 (32)	4 (27)
- WOUND INFECTION, N (%)	3 (6)	2 (5)	1 (7)
**PRESSURE ULCERS, N (%)**	10 (19)	8 (22)	2 (13)
**MR IMAGING PRE-OP, N (%)**	49 (94)	35 (95)	14 (93)
**TIME IN NEUROCRITICAL CARE, DAYS, MEDIAN (IQR)**	5(5)	5(5)	6(5)

**Fig. 2:**
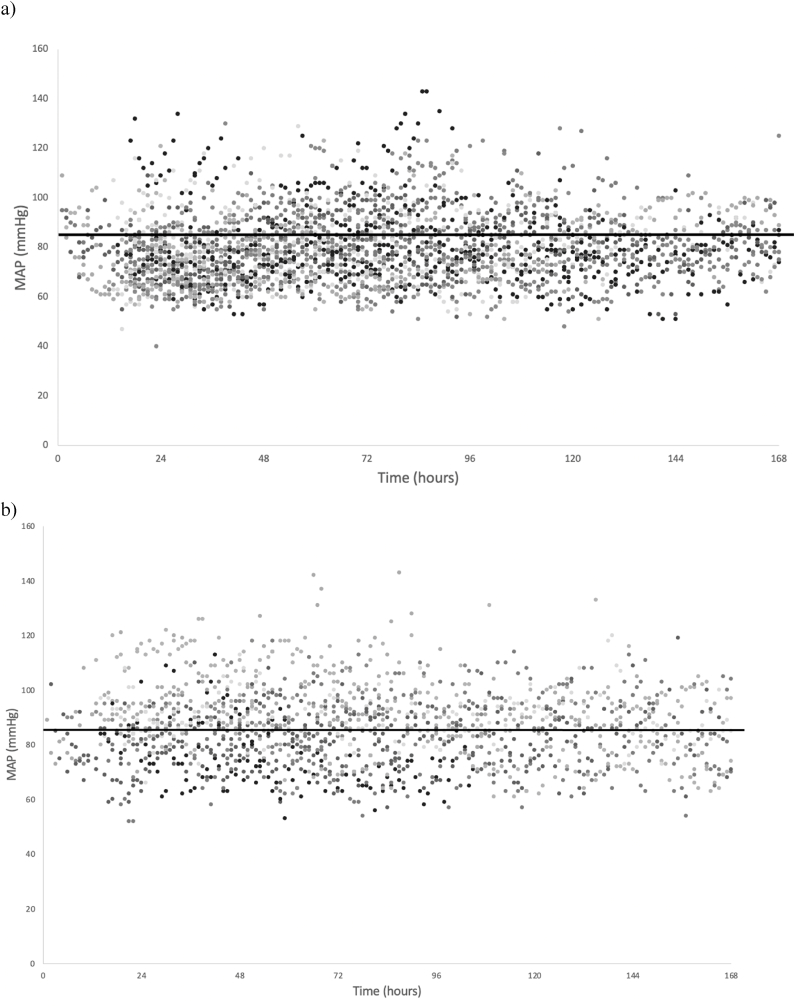
Hourly MAP values during the initial seven days after injury. (a) MAP values before 2013. (b) MAP values after 2013. Horizontal line showing the guidelines MAP target of 85 mmHg. MAP values were significantly higher after 2013 though a large proportion failed to meet the MAP target.

MAP values during the initial seven days after injury are illustrated in Fig. 2. The mean MAP was significantly higher after 2013. In 69% of patients before 2013, MAP thresholds set by current guidelines were not met, compared to 44% after 2013 (p < 0.05). In total, 62% of all patients had >50% of recorded MAP values below 85 mm Hg. Only a few values of systolic blood pressures below 90 mmHg were recorded, regardless of the evaluated period. Mean systolic blood pressure increased slightly with time, though without reaching statistical significance (123±17.2 mmHg compared to 131±15.2 mmHg before and after 2013 respectively, p = 0.11). Crystalloids were administered to 88% of patients, and colloids and blood to 42% of the patients, mainly to improve blood pressure. Dobutamine (31%) was more often used as an ionotropic treatment than norepinephrine (21%). Oxygen saturation was above 92% in most patients and mean saturation was unaffected by the implementation of guidelines, (97.3±0.2%) prior to 2013 and (97.5±0.3%) after 2013.

**Fig. 3:**
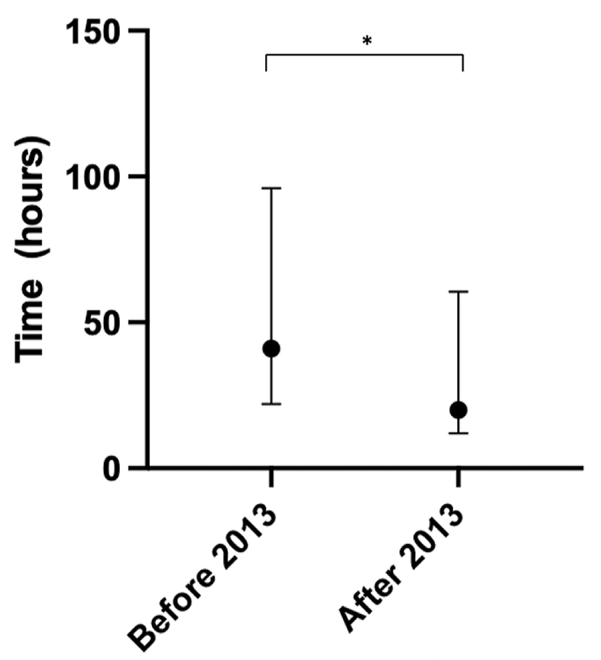
Median (iqr) time to surgery before and after 2013. The median time to surgery was significantly shorter after 2013.

Median time to surgery was significantly shorter after 2013 (20 h, compared to 41 h before 2013, p < 0.05, Fig. 3). Before 2013, 62% were operated after 24 h *vs.* 33% after 2013. Pre-operative magnetic resonance imaging (MRI) was performed in 49 (94%) cases without change between the periods. Posterior approaches were used in most cases (55%) before 2013. After 2013 anterior and posterior approaches were equally common (38% each). The remaining 24% had a combination of anterior and posterior approaches.

Urinary tract infection (UTI) was the most common complication, seen in 21 (41%) patients. The occurrence of UTI increased after 2013 (53% compared to 35% prior to 2013). Pneumonia occurred in 16 (31%) patients, wound infection in three (6%) patients, and pressure ulcers in 10 (19%) patients. The occurrence of pneumonia decreased from 32% to 27% and pressure ulcers from 22% to 13% after 2013. Fever >38 °C was recorded in 624 h, >39 °C for 128 h, and >40 °C for 19 h of a total of 4343 h monitored. Before 2013, temperatures >38 °C were noted in 488 h compared to 283 h after 2013.

Five patients (14%) suffered VTE events before 2013 compared to one patient (7%) after 2013, of whom deep venous thrombosis occurred in three (6%) and pulmonary embolism in three patients (6%). The most prescribed thromboembolic prophylaxis was compression stocking in combination with enoxaparin (36%). The mean time until initiation of venous thromboembolic prophylaxis was 6 (±4) days before 2013 and 4 (±3) days after 2013. The time to initiation of thromboembolic prophylaxis was significantly longer in patients who suffered a thromboembolic event (eight days compared to four days, p = 0.03).

**Fig. 4:**
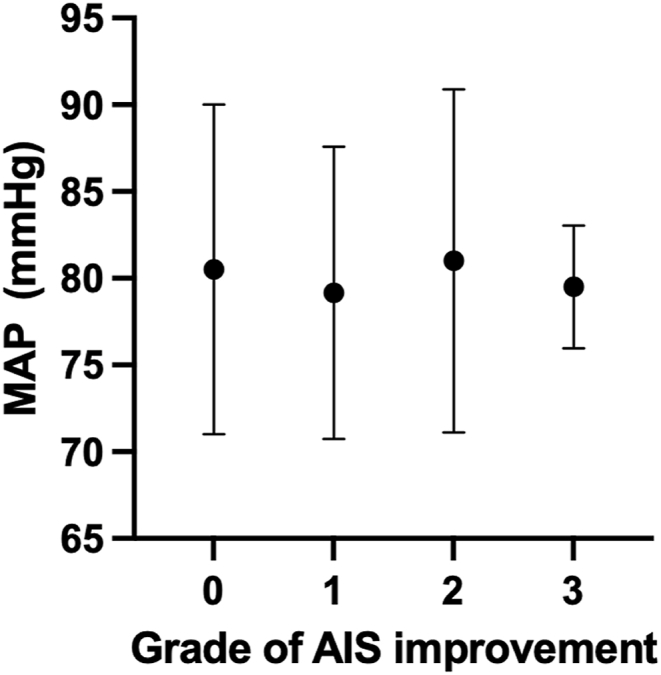
Mean (SD) MAP divided by AIS grade of improvement. There were no significant differences in MAP based on neurological improvement.

No AIS grade improvement was seen in 30 patients (58%). Six patients (12%) had one AIS grade of improvement, two patients (4%) improved two AIS grades and two patients (4%) improved three AIS grades. In 12 patients (23%), no information on neurological outcome was available. No significant difference in mean MAP was seen between the four groups of AIS improvement (Fig. 4).

There was a significantly larger spinal cord anterior-posterior diameter on pre-operative MRI in groups with a higher level of AIS grade improvement, p = 0.048. The length of the spinal cord edema was not different between the groups of AIS grade improvement.

## 4 Discussion

In the present study, we evaluated the neurocritical care management of patients with acute traumatic SCI before and after the implementation of management guidelines published in 2013. All patients had cervical injuries and the vast majority had AIS grade of A-B, i.e. motor complete injuries. The major findings were that after the introduction of the 2013 guidelines, higher MAP values were noted, even though only a minority of all measured values met the MAP threshold suggested by the guidelines. Additionally, surgery was performed earlier, and the time to VTE prophylaxis was shorter. The implementation of guidelines did not, however, translate into improved neurological outcome in our patient cohort.

A recent study reports significant neurological improvement by MAP kept ≥85 mmHg (Hawryluk et al., 2015). In the present cohort, MAP values were significantly higher after the revised 2013 guidelines, recommending MAP above 85 mmHg during the initial seven days after injury (Ryken et al., 2013), were implemented. However, we could not verify a significant association between MAP and improved neurological outcome. Likely, several factors account for these results. Firstly, a subset of patients was lost to follow up and the time to surgical decompression remained long even after the guidelines were introduced. In addition, despite increased awareness of the importance of maintaining MAP, in 44% of the patients the MAP target was not met after 2013. Although all patients were treated in the neurocritical care unit, the loss of sympathetic tone observed in cervical SCI may have contributed to the difficulties in achieving a sufficient MAP. Since the pathophysiology of SCI is complex, improved spinal cord perfusion by an increased MAP may not compensate for all secondary injury mechanisms (Kwon et al., 2024; Evaniew et al., 2020). Finally, MAP may not correctly reflect spinal cord perfusion pressure, as we did not monitor intraspinal pressure (Saadoun and Papadopoulos, 2016). Ongoing, as well as future studies will reveal the effect of neurointensive monitoring and duroplasty on SCI outcome (Saadoun and Papadopoulos, 2021; Saadoun et al., 2023). Our results argue for an increased awareness of the importance of MAP and adherence to MAP targets when implementing guidelines.

Guidelines also recommend early decompressive surgery. The interest in early or even ultra-early decompression of the spinal cord in SCI patients is increasing even though “early” and “ultra-early” have not been defined. Findings from the STASCIS trial, showing a two-grade AIS improvement in 20% of the patients having surgery within 24 h of SCI compared to 9% in the group of late intervention, and a recent meta-analysis support the clinical benefit of early surgery (Fehlings et al., 2012; Badhiwala et al., 2021). Another study argues that the ultimate time for surgery is within the first 4–9 h after injury, resulting in an higher rate of ≥ two AIS grade improvement at 6 months post-injury (Jug et al., 2019). Mattiassich et al., in contrast, failed to show an improved outcome with surgery performed within 5 h post-injury (Mattiassich et al., 2017). In addition, the recent SCI-POEM study comparing surgery ≤12 h after injury to surgery beyond 12 h in 294 patients, failed to show improvement at 12 months with early surgery (Hosman et al., 2023). It is speculated that the dynamic and unstable initial hemodynamic and neurological situation, in addition to logistical challenges, may outweigh the benefits of ultra-early surgical decompression since the patient may be exposed to a greater risk with surgery during the initial phase (Mattiassich et al., 2017). To date, the best available evidence suggests benefits of early surgical decompression, defined as < 24h post-injury (Ter Wengel et al., 2019). While the timing of surgical intervention was shorter in our department after implementing the guidelines, early surgery was still performed beyond 24 h post-injury in 33% of cases. Worth noting is that our cohort presents with high-grade AIS injuries. More than 67% presented with motor complete injuries, compared to 32% in the STASCIS study (Fehlings et al., 2012) making comparisons difficult. We cannot preclude that delayed surgery clouds the effect of other changes in the implementation of guidelines.

There is no pharmacological treatment option available to SCI patients. We observed a robust reduction in the use of high-dose methylprednisolone after 2013 which is in line with current recommendations (Walters et al., 2013). Presumably more important in SCI care is that the implementation of guidelines likely results in a stronger emphasis on neurocritical care management. We observed, after 2013, a decrease in early complications such as pressure ulcers and pneumonia although not in urinary tract infections. Moreover, the current recommendation is to initiate VTE prophylaxis within 72 h post-injury (Christie et al., 2011). We note earlier initiation of prophylaxis after guideline implementation, though not within the recommended 72-h time frame. Encouragingly, the incidence of thromboembolic events decreased after 2013. It is unlikely to initiate prophylactic treatment before surgery, and delayed surgery is associated with delayed VTE prophylaxis. In previous studies, early initiation of VTE prophylaxis after SCI reduced the incidence of thromboembolic events from 26% to 2% of the patients in patients with delayed initiation of prophylaxis (Harris et al., 1996; Aito et al., 2002).

The retrospective design of this study has inherent limitations, e.g. leading to some patients being lost to follow-up. Also, the follow-up time was short (three months). In many cases further improvement, up until 6–9 months is possible (Eckert and Martin, 2017). Utilizing guidelines as a care bundle does improve MAP values, promote early surgical decompression, and initiation of antithrombotic treatment. The goals of the guidelines are intertwined, e.g. delayed surgery leads to longer time with compression of the spinal cord which in turn likely aggravates the secondary injury, reducing the effect of improved spinal cord perfusion. Also, delayed surgery postpones the initiation of antithrombotic prophylactic treatment. The present study shows how key neurocritical care parameters were altered after implementing guidelines. Our main aim was to review the effect of the different treatment goals stipulated by the guidelines. We did not, however, aim to review the effects on clinical outcome after the different treatment goals were altered, for which larger, multicenter approaches are needed. Such comparisons on outcome would likely be aided by propensity score matching given the low number and heterogeneity of SCI patients. Given the aim of our study, such statistical methods would thus not change the main conclusions. There is obvious room for improvement in the adherence to the goals of the guidelines in our present study, which in the future might aid in improving neurological outcome amongst SCI patients.

## 5 Conclusion

Using guidelines to reach treatment endpoints for SCI is likely effective in maintaining cohesive goals of care. Improving one endpoint likely leads to improvement in others. In the present paper, exploring the effects of guidelines implementation, the thresholds set by SCI guidelines were insufficiently met and no improvement in patient neurological outcome was noted. Further stringent adherence to guidelines is warranted for future improvement in patient outcome.

## Conflict of interest

N.M. is a scientific advisor for Polarcool Inc, Lund, and Teqcool Lund, Sweden, of no relevance to the present study. The authors declare no other conflict of interest.

## Funding

Hospital funds provided by Skane University Hospital, Lund.

## 6 Contributions

All authors have made substantial contributions to the present article. Conceptualization and design (FR, NM). Acquisition of data (FR). Analysis and interpretation of data (FR, EU, NM). Drafting the article (FR). Text revision (FR, EU, NM). Final approval of the version submitted (FR, EU, NM)

## Declaration of competing interest

The authors declare the following financial interests/personal relationships which may be considered as potential competing interests:

Niklas Marklund reports a relationship with Polarcool Inc that includes: consulting or advisory. Niklas Marklund reports a relationship with Teqcool that includes: consulting or advisory. If there are other authors, they declare that they have no known competing financial interests or personal relationships that could have appeared to influence the work reported in this paper.
